# Roles of Exogenous α-Lipoic Acid and Cysteine in Mitigation of Drought Stress and Restoration of Grain Quality in Wheat

**DOI:** 10.3390/plants10112318

**Published:** 2021-10-28

**Authors:** Amr Elkelish, Mohamed M. El-Mogy, Gniewko Niedbała, Magdalena Piekutowska, Mohamed A. M. Atia, Maha M. A. Hamada, Mostafa Shahin, Soumya Mukherjee, Ahmed Abou El-Yazied, Mohamed Shebl, Mohammad Shah Jahan, Ali Osman, Hany G. Abd El-Gawad, Hatem Ashour, Reham Farag, Samy Selim, Mohamed F. M. Ibrahim

**Affiliations:** 1 Botany Department, Faculty of Science, Suez Canal University Ismailia, Ismailia 41522, Egypt; amr.elkelish@science.suez.edu.eg; 2 Vegetable Crops Department, Faculty of Agriculture, Cairo University, Giza 12613, Egypt; 3 Department of Biosystems Engineering, Faculty of Environmental and Mechanical Engineering, Poznań University of Life Sciences, Wojska Polskiego 50, 60-627 Poznań, Poland; gniewko.niedbala@up.poznan.pl; 4 Department of Geoecology and Geoinformation, Institute of Biology and Earth Sciences, Pomeranian University in Słupsk, Partyzantów 27, 76-200 Słupsk, Poland; magdalena.piekutowska@apsl.edu.pl; 5 Molecular Genetics and Genome Mapping Laboratory, Genome Mapping Department, Agricultural Genetic Engineering Research Institute (AGERI), Agricultural Research Center (ARC), Giza 12619, Egypt; matia@ageri.sci.eg; 6 Department of Agronomy, Faculty of Agriculture, Ain Shams University, Cairo 11566, Egypt; Maha_hamada@agr.asu.edu.eg (M.M.A.H.); mostafa_shahin@agr.asu.edu.eg (M.S.); 7 Department of Botany, Jangipur College, University of Kalyani, Kalyani 742213, India; soumobios@gmail.com; 8 Department of Horticulture, Faculty of Agriculture, Ain Shams University, Cairo 11566, Egypt; ahmed_abdelhafez2@agr.asu.edu.eg (A.A.E.-Y.); hany_gamal2005@agr.asu.edu.eg (H.G.A.E.-G.); 9 Food Science Department, Faculty of Agriculture, Ain Shams University, Cairo 11566, Egypt; Mohamed6080@agr.asu.edu.eg; 10 College of Horticulture, Nanjing Agricultural University, Nanjing 210095, China; shahjahansau@gmail.com; 11 Department of Horticulture, Sher-e-Bangla Agricultural University, Dhaka 1207, Bangladesh; 12 Biochemistry Department, Faculty of Agriculture, Zagazig University, Zagazig 44511, Egypt; aokhalil@zu.edu.eg; 13 Department of Agricultural Botany, Faculty of Agriculture, Ain Shams University, Cairo 11566, Egypt; Hatem_ashour@agr.asu.edu.eg (H.A.); Reham_hassan@agr.asu.edu.eg (R.F.); 14 Department of Clinical Laboratory Sciences, College of Applied Medical Sciences, Jouf University, Sakaka 72388, Saudi Arabia; sabdulsalam@ju.edu.sa

**Keywords:** wheat, water stress, antioxidant capacity, grain quality, alveographic parameters, alpha-lipoic acid, cysteine

## Abstract

Cysteine (Cys) and α-lipoic acid (ALA) are naturally occurring antioxidants (sulfur-containing compounds) that can protect plants against a wide spectrum of environmental stresses. However, up to now, there are no conclusive data on their integrative roles in mitigation of drought stress in wheat plants. Here, we studied the influence of ALA at 0.02 mM (grain dipping pre-cultivation treatment) and Cys (25 and 50 ppm as a foliar application) under well watered and deficit irrigation (100% and 70% of recommended dose). The results showed that deficit irrigation markedly caused obvious cellular oxidative damage as indicated by elevating the malondialdehyde (MDA) and hydrogen peroxide content (H_2_O_2_). Moreover, water stressed plants exhibited multiple changes in physiological metabolism, which affected the quantitative and qualitative variables of grain yield. The enzymatic antioxidants, including superoxide dismutase (SOD), ascorbate peroxidase (APX), catalase (CAT) and peroxidase (POX) were improved by Cys application. SOD and APX had the same response when treated with ALA, but CAT and POX did not. Moreover, both studied molecules stimulated chlorophyll (Chl) and osmolytes’ biosynthesis. In contrast, the Chl a/b ratio was decreased, while flavonoids were not affected by either of the examined molecules. Interestingly, all above-mentioned changes were associated with an improvement in the scavenging capacity of reactive oxygen species (ROS), leaf relative water content (RWC), grain number, total grain yield, weight of 1000 kernels, gluten index, falling number, and alveographic parameters (P, W, and P/L values). Furthermore, heatmap plot analysis revealed several significant correlations between different studied parameters, which may explore the importance of applied Cys and ALA as effective compounds in wheat cultivation under water deficit conditions.

## 1. Introduction

Wheat (*Triticum aestivum* L.) is an extremely important cereal crop for human nutrition and animal feed worldwide. Though wheat cultivation is pervasive in a wide spectrum of ecosystems, many parts of the world (particularly in the arid and semiarid regions) suffer from drought stress as a major constraint hindering its expansion from meeting the growing demands day by day for the population [1,2].

Frequent climate change scenarios and rareness of fresh water are two major limiting constraints to sustainable agriculture and preserving food security worldwide. These two major problems may be exacerbated in the semiarid regions where the annual precipitation (200 to 750 mm/year) is not sufficient to meet the needs for farming throughout the year [3,4]. In plants, drought stress or water deficit can trigger a wide spectrum of successive events at morphological, biochemical, and molecular levels that may contribute to the adaptation or tolerance processes to such conditions [5,6,7,8]. Among these responses is the excessive release of reactive oxygen species (ROS) that induce oxidative damage to plant cell components, i.e., protein, lipids and, nucleic acids [9,10,11], and the development of several efficient non-enzymatic and enzymatic antioxidant systems [1,12,13]. Plant resilience to drought stress is mostly attained by accumulating compatible solutes, ion homeostasis, and redox management [5,14,15,16]. Additionally, water shortage can trigger a cascade of interconnected responses that coordinate the physiological metabolisms of plants, such as altering the signaling pathways, gene expression, hormonal homeostasis, and photosynthetic machinery, leading eventually to affecting crop productivity [8,17,18,19,20,21].

Sulfur-containing molecules and a number of non-protein and protein thiols have been evidenced to play a fundamental role in plant tolerance to various abiotic stresses [22,23,24]. These molecules can work together, representing a crucial network of responses that enable plants to cope with environmental stresses [24]. Therefore, special attention should be given to reveal a complete picture in this respect.

Alpha-lipoic acid (1,2-dithiolane-3-pentanoic acid; ALA) is a dithiol short-chain fatty acid that is ubiquitous both in prokaryotic or eukaryotic organisms [25]. It has received great attention as a dietary supplement for humans because of its antioxidant and therapeutic properties [26,27]. This antioxidant capacity depends on its two sulfhydryl moieties [25] which enable it to scavenge free radicals and chelate metals [28]. In cereal crops, a few studies during the seedling stage have reported that exogenous ALA can enhance the photosynthetic performance in maize under drought stress [29], ameliorate lipid peroxidation, and induce the antioxidant systems of maize under osmotic stress [25]. Furthermore, it can regulate the ion homeostasis and osmotic potential in wheat under saline conditions [30]. In addition, it has been found that there is a close connection between the endogenous ALA and the cellular redox status of wheat seedlings grown under excess copper [31].

Cysteine (Cys) is an essential thiol-containing amino acid that comprises amino group (NH_2_), carboxylic acid (COOH) and sulfhydryl group (SH) as reactive centers. This distinct structure enables Cys to be a potent antioxidant and efficient scavenger for reactive oxygen species (ROS). This is due to the presence of the thiol side chain, which smoothly oxidizes, protecting against the oxidative damage induced by biotic and abiotic stresses [32,33]. Moreover, Cys is involved in synthesizing a wide array of vital and defensive molecules such as protein, glutathione, phytoalexins, thionins, glucosinolates, metallothioneins, and phytochelatins [23,32,34,35,36]. Additionally, Cys is implicated in different sulfur metabolism pathways and the synthesis of methionine [33]. These responses can regulate plant growth and development via the importance of methionine and/or S-adenosylmethionine (SAM) as precursors for some vital phytohormones, i.e., ethylene and polyamines [37,38]. Several lines of evidence have suggested that exogenous Cys can alleviate the deleterious effects of heavy metals in plants through its roles in metal sequestration and detoxification [35,36]. Similarly, applied Cys has been shown to delay the senescence of postharvest green leafy vegetables by decreasing the rate of Chl degradation, cellular respiration, and ethylene biosynthesis [39]. Up to now, there are no conclusive data to elucidate the role of ALA and/or Cys in wheat plants subjected to water deficit. The current research explores novel approaches to learn more about the combined effect of biomolecules on the diverse parameters of wheat plants under water deficit.

## 2. Materials and Methods

### 2.1. Plant Material, Treatments, and Experimental Design

A field experiment was conducted during the period from November to April in 2018/2019 and 2019/2020 at a private farm at Beheira Governorate, Egypt (latitude: 30.8481 N; longitude: 30.34 E; mean altitude: 60 m above sea level). Healthy and uniform wheat grains (*Triticum aestivum* L, Cv. Giza 168) were divided into two groups. The first group of grains was soaked in distilled water for 4 h and then sown in the experimental soil, whereas the second group was soaked for 4 h in a solution containing 0.02 mM α-lipoic acid (ALA, Cayman Chemical Company, Michigan, USA). The experimental design was Split-Split-Plot arranged into a randomized complete block design (RCBD) with three replicates. Irrigation with two application levels of 70% and 100% of the recommended dose for wheat plants was set up in the main plots, while ALA-treated and non-treated grains were distributed in the sub plots. Cys foliar applications (Cys, Alpha Chemika, Mumbai, India) at 0, 25 and 50 ppm plus 0.05% (*v*/*v*) Tween 20 as a non-ionic surfactant were randomly distributed in the experimental units. The experimental unit was 15 m^2^ (3 × 5 m) with sowing space 12 cm. Applied Cys was repeated three times during the growing season, starting from the tillering to stem extension stage at 25, 45, and 65 days after sowing. At the anthesis stage (85 days after sowing), leaves were collected for different biochemical estimations, whereas the total yield (ton·ha^−1^), various physical/chemical properties and alveographic parameters of grains were determined at the end of each season (158 days after sowing).

### 2.2. Irrigation Requirements

A local weather station registered meteorological data. The mean daily temperature data, wind speed at the height of 2 m, precipitation, daily solar radiation, and relative humidity were used in developing the Reference Evapotranspiration (ET_o_) in mm/day. The ET_o_ was created according to the calculation procedure given in FAO paper n. 56 [40]. Estimation of irrigation requirement (IR) in m^3^/ha is derived from crop evapotranspiration (ETc), which can be calculated by multiplying the ET_o_ with the crop coefficient (Kc). It has been taken into account that the sprinkler irrigation efficiency is 75% when calculating the IR. Based on the quantities of IR calculated in Table 1, the irrigation quantities in the main experimental plots were determined with two application levels of 70% and 100% from IR. The period considered in this study was calculated ET_o_ for two consecutive seasons (2018/2019 and 2019/2020). The ET_o_ equation can be expressed as:

(1) ETo=0.408 Δ Rn−G+γ 900T+273U2es−eaΔ+1+0.34 U2

where Δ is the slope of the vapor pressure curve (kPa·°C^−1^), R_n_ the surface net radiation (MJ·m^−2^·day^−1^), G soil heat flux density (MJ·m^−2^·day^−1^), γ psychometric constant (kPa·°C^−1^), T mean daily air temperature (°C), U_2_ wind speed (m·s^−1^), *e_s_* saturated vapor pressure, *e_a_* actual vapor pressure.

Kc = Crop Coefficient, ET_o_ = Reference Evapotranspiration (mm), IR = Irrigation Requirements (m^3^/ha). (2)

### 2.3. Agricultural Management

Wheat grains were sown using 178.57 kg ha^−1^ at the 10th and 8th of November in the first and second season, respectively. The recommended doses of mineral fertilizers (NPK) were applied as follows: P (36 kg ha^−1^) was supplemented during the soil preparation as calcium super phosphate 15.5% P_2_O_5_, while N (187.5 kg ha^−1^) was added as ammonium nitrate (33.5% N) into seven equal portions, at 10, 20, 30, 40, 50, 60 and 70 days after sowing. Additionally, K (79.2 kg ha^−1^) was supplemented as potassium sulfate (48% K_2_O) in two equal portions at sowing and heading stages.

### 2.4. Determination of Chlorophyll (Chl)

The leaf content of Chl a and Chl b was determined as described by Costache et al. [41] with some modification, small pieces of fresh leaves (0.5 g) were submerged into 10 mL pure acetone for 24 h/4 °C in small dark bottles to avoid pigment degradation. The homogenate was centrifuged at 4000 rpm for 15 min then the absorbance was measured at 645 and 663 nm, respectively. The concentration was calculated using the following equations:

Chl a (mg/g FW) = 11.75 A_662_ − 2.350 A_645_ × (V/1000 × W) (3)

Chl b (mg/g FW) = 18.61 A_645_ − 3.960 A_662_ × (V/1000 × W) (4)

where, A is the absorbance at 645 and 663 nm, V is the final volume of Chl extract in pure acetone and W is the fresh weight of tissue extract. Additionally, Chl a+b and Chl a/b ratio were calculated.

### 2.5. Determination of Osmolytes and Leaf Water Status

Proline concentration was determined with ninhydrin reagent as described by Bates et al. [42]. Total soluble sugars were estimated by phenol-sulfuric acid method as described by Chow and Landhäusser [43]. Leaf relative water content was determined according to Ünyayar et al. [44]. Leaf discs from 10 leaves were weighed (FW) and placed immediately in distilled water for 2 h at 25 °C and then their turgid weights (TW) were recorded. The samples were then dried in an oven at 110 °C for 24 h (DW). Relative water content (RWC) was calculated by using the following formula: RWC = (FW − DW)/(TW − DW) × 100.

### 2.6. Determination of Oxidative Damage and Scavenging Capacity

The level of lipid peroxidation was measured by the determination of malondialdehyde (MDA) as described by Heath and Packer [45]. One gram of fresh leaves was homogenized in 10 mL diluted trichloroacetic acid (TCA; 0.1% *w*/*v*). The homogenate was centrifuged at 4500 rpm for 15 min. The reaction mixture contained 1 mL from the supernatant and 4 mL 0.5% (*w*/*v*) thiobarbituric acid (TBA) dissolved in 20% (*w*/*v*) TCA. The mixture was heated in boiling water for 30 min then the mixture was cooled at room temperature and centrifuged at 4500 rpm for 15 min. The absorbance of the supernatant was measured at 535 nm and corrected for non-specific turbidity at 600 nm using a spectrophotometer (UV-1601PC; Shimadzu, Tokyo, Japan). The MDA concentration (nmol·g^−1^ FW) was calculated using Δ OD (A532-A600) and the extinction coefficient (ε = 155 mm^−1^ cm^−1^).

Hydrogen peroxide (H_2_O_2_) concentration was determined according to Velikova et al. [46] with some modifications. Leaf samples of 0.5 g were homogenized in 3 mL of 1% (*w*/*v*) tri-chloroacetic acid (TCA). The homogenate was centrifuged at 10,000 rpm and 4 °C for 10 min. Subsequently, 0.75 mL of the supernatant was added to 0.75 mL of 10 mM K-phosphate buffer (pH 7.0) and 1.5 mL of 1M KI. H_2_O_2_ concentration was evaluated by comparing its absorbance at 390 nm to a standard calibration curve. The concentration of H_2_O_2_ was calculated from a standard curve plotted in the range from 0 to 15 nmol mL^−1^.

The scavenging capacity of free radicals was estimated by the reduction of the reaction color method of 1,1-Diphenyl-2-picrylhydrazyl (DPPH) with sample extract as described by Huang et al. [47]. One gram of fresh leaves was homogenized in 20 mL of 70% methanol (*v*/*v*) for 15 h and kept in dark until assay. A final concentration (0.15 mM) of DPPH solution (3.9 mL) was mixed with sample solution (0.1 mL). The mixture was kept in the dark at ambient temperature. The absorbance of the mixtures was recorded at 515 nm for exactly 30 min. Blank was made from 3.9 mL of DPPH and 0.1 mL methanol and measured absorbance at T_0_. The scavenging of DPPH was calculated according to the following equation:

% DPPH scavenging = [(Abs T_0_ − Abs T_30_)/Abs T_0_] × 100 (5)

where Abs (T_0_) = absorbance of DPPH at 0 time, T_30_ absorbance at 30 min.

### 2.7. Determination of Non-Enzymatic Antioxidants

Carotenoids were quantified using the acetone and petroleum ether method as described by de Carvalho et al. [48] using the following formula:

Carotenoids (mg/g FW) = A_450_ × V (mL) × 10/[A^1%^_1cm_ × W (g)] (6)

where A_450_ = absorbance at 450 nm, V = total extract volume; W = sample weight; A^1%^_1cm_ = 2592 (β-carotene coefficient in petroleum ether). The specific wavelengths for all estimated leaf pigments were determined using UV visible spectrophotometer (UV-1601PC; Shimadzu, Tokyo, Japan).

Aluminum chloride colorimetric method was used for flavonoids determination [49]. Each plant was extracted (0.5 mL of 1:10 g mL^−1^) in methanol and was separately mixed with 1.5 mL of methanol, 0.1 mL of 10% aluminum chloride, 0.1 mL of 1 M potassium acetate, and 2.8 mL of distilled water. It was kept at room temperature for 30 min; the reaction mixture’s absorbance was measured at 415 nm. The calibration curve was obtained by preparing quercetin solutions at concentrations of 12.5 to 100 µg/mL.

The extraction of fresh leaves in cold MeOH 85% was used to determine total soluble phenols according to the method of Folin–Denis as described by Shahidi et al. [50]. One milliliter of crude extract was mixed with 0.5 mL of Folin–Denis reagent and were well mixed in dry test tube, the tube was thoroughly shaken for 3 min, 1.0 mL of saturated Na_2_CO_3_ solution was added and well mixed then 3 mL of distilled water was added. After one hour, phenolic compounds were determined by reading the developed blue color at 725 nm by using a spectrophotometer. Ascorbic acid (AsA) was determined using 2, 6-Dichloroindophenol titrimetric method [51].

### 2.8. Determination of Antioxidant Enzymes Activity

To prepare the extraction of enzyme and soluble proteins, fresh leaves (0.5 g) were homogenized in 4 mL 0.1 M sodium phosphate buffer (pH 7.0) containing 1% (*w*/*v*) polyvinylpyrrolidone (PVP) and 0.1 mM EDTA, centrifuged at 10,000× *g* for 20 min at 4 °C and then the supernatant was used for assays. Soluble proteins were evaluated by the method of Bradford [52]. All studied enzyme activities and protein concentration in the crude enzyme extract were measured using a spectrophotometer (UV-1601PC; Shimadzu, Tokyo, Japan) as follows.

Superoxide dismutase (SOD) assay was based on the method described by Beyer et al. [53]. The reaction mixture with a total volume of 3 mL contained 100 μL crude enzyme, 50 mM phosphate buffer (pH 7.8), 75 μM NBT, 13 mM L-methionine, 0.1 mM EDTA and 0.5 mM riboflavin. The addition of riboflavin initiated the reaction then the reaction mixture was illuminated for 20 min with a 20 W fluorescent lamp. One enzyme activity unit was defined as the amount of enzyme required to result in a 50% inhibition in the rate of nitro blue tetrazolium (NBT) reduction at 560 nm.

Catalase (CAT) activity was measured by monitoring the decrease in absorbance at 240 nm as described by Cakmak et al. [54]. The reaction mixture with a total volume of 3 mL contained 15 mM H_2_O_2_ in 50 mM phosphate buffer (pH = 7). The reaction was initiated by adding 50 μL crude enzyme. The activity was calculated from the extinction coefficient (ε = 40 mm^−1^ cm^−1^) for H_2_O_2_. One unit of enzyme activity was defined as the decomposition of 1 μmol of H_2_O_2_ per minute.

The activity of ascorbate peroxidase (APX) was determined according to Nakano and Asada [55]. The decrease of absorbance at 290 nm was monitored for 3 min. The reaction mixture with a total volume of 3 mL included 100 µL crude enzyme, 50 mM phosphate buffer (pH 7), 0.1 mM EDTA, 0.5 mM ascorbic acid, and 0.1 mM H_2_O_2_. The addition of H_2_O_2_ initiated the reaction. One enzyme activity unit was defined as the amount of enzyme required for oxidation of 1 µmol of ascorbate per minute. The rate of ascorbate oxidation was calculated using the extinction coefficient (ε = 2.8 mm^−1^ cm^−1^).

Peroxidase (POX) activity was quantified by the method of Dias and Costa [56] with some minor modifications. The assay mixture (100 mL) contained 10 mL of 1% (*v*/*v*) guaiacol, 10 mL of 0.3% H_2_O_2_ and 80 mL of 50 mM phosphate buffer (pH = 6.6). The volume of 100 µL of the crude enzyme was added to 2.9 mL of the assay mixture to start the reaction. The absorbance was recorded every 30 s for 3 min at 470 nm.

### 2.9. Properties of Wheat Grains

#### 2.9.1. Physical/Chemical Characteristics

Moisture was determined according to AACC standard method No 44-15.02 [57]. Weight per 1000 kernels and hectoliter weight were determined according to AACC standard method No 55-10.01 [57]. Wet gluten and gluten index were determined for wheat meal. Ground whole grain was performed by laboratory mill (3100 with 0.8-mm screen). Wet gluten was washed from whole-grain wheat meal by an automatic gluten washing apparatus (Glutomatic system 2200, Perten Instruments AB, Huddinge, Sweden). Then, it was centrifuged on a specially constructed sieve under standardized conditions. The weight of wet gluten was forced through the sieve then total wet gluten was measured according to AACC standard method No 38-12.01 [57]. The total wet gluten was expressed as percent of sample, and the gluten index was expressed as percentage of wet gluten remaining on the sieve after centrifuging. Hagberg falling number was determined according to AACC standard method No 56-81.03 [57] using FN 1500 (Perten Instruments AB, Hagersten, Sweden). This method is based on the ability of α-amylase to liquefy a starch gel. The activity of the enzyme is measured by falling number (FN), defined as time in sec required to stir and allow stirrer to fall a measured distance through a hot aqueous flour or meal gel undergoing liquefaction. α-Amylase activity is associated with kernel sprouting, and both of these are inversely correlated with FN. Protein (N×5.7) content was analyzed according to AOAC standard method No 920.87 [58].

#### 2.9.2. Alveograph Test

Alveographic properties were evaluated using Chopin Alveograph (Chopin, Villeneuve La Garenne cedex—France) according to AACC standard method No 54-30.02 [57]. Alveograph test was carried out to determine resistance of dough to extension (maximum overpressure, *P*; this is average of maximum ordinates, measured in mm), extensibility (average abscissa at rupture, *L*; abscissa at rupture of each curve is measured in mm on zero line, from origin of curve to point corresponding vertically with clear drop due to rupture of bubble; average of abscissae at rupture points of curves, expressed to nearest unit, represents length), curve configuration ratio, *P*/*L*, and Deformation energy of dough, *W*. Average curve is drawn based on average of ordinates up to *L*, expressed in 10^−4^ J.

### 2.10. Statistical Analysis and Data Visualization

A combined analysis of variance over the two seasons was carried out (Table 2). The significance of difference among the studied varieties was tested by analyzing variance (ANOVA) test as outlined by Snedecor and Cochran [59]. Mean comparisons for variables were made among genotypes using least significant differences (LSD at 5%) tests. Moreover, silhouette analysis was performed to evaluate the quality of the irrigation, seed soaking, and foliar application treatments measurements clustering by testing the cluster distances within and between each cluster [60]. Additionally, we performed a multidimensional preference analysis to disclose the interrelationships amongst parameters in addition to the similarity classification of in terms of dependent and independent variables [61]. Finally, hierarchical clustering based on the correlation analysis was conducted and two-dimensional heatmap plotting was constructed. Boxplot of the parameter’s classes and “seed soaking and foliar application” levels are represented graphically, showing distribution of the data, and the X inside the box represents the mean. Colored Mosaic plot was displayed to summarizing the high dimensional data levels. The heatmap, Mosaic plot and Boxplot were drawn with R software.

## 3. Results

### 3.1. Changes in Chlorophyll Composition

**Figure 1:**
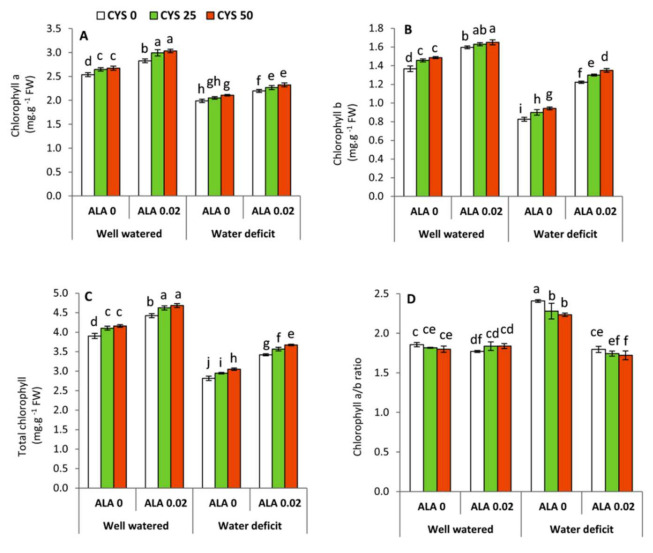
Effect of irrigation level, seed soaking by α-lipoic acid (ALA; 0 and 0.02 mM) and foliar application by cysteine (Cys; 0, 25 and 50 ppm) on the chlorophyll concentration of wheat plants. (**A**) chlorophyll a, (**B**) chlorophyll b, (**C**) total chlorophyll, (**D**) chlorophyll a/b ratio. For each parameter, the mean values ± SD followed by a different letter are significantly different according to Tukey’s range test (*p* ≤ 0.05).

Analysis of variance (Table 2) shows that all leaf chlorophyll compositions including Chl a, Chl b, Chl a+b, and Chl a/b ratio were dramatically and significantly affected by irrigation level (I), ALA as seed soaking (S) and Cys as foliar application (F) treatments. However, the interaction between the irrigation level and seed soaking (I × S) was significant in respect to the Chl a, Chl a+b and Chl a/ b ratios. Furthermore, the Chl a/b ratio was significantly affected by the interaction treatments of I × F and S × F. Generally, deficit irrigation caused an obvious and significant (*p* ≤ 0.05) decrease in Chl a, Chl b and Chl a+b, whereas the Chl a/b ratio was significantly increased particularly in the plants not treated with ALA (Figure 1). This trend indicates the positive effect of ALA as a seed soaking treatment on maintaining the content of Chl b under water shortage. The highest significant increase in Chl a, Chl b and Chl a+b was obtained by the treatment of 0.02 mM ALA + 50 ppm Cys under well watered conditions. In contrast, under limited water supply, the treatments of 0.02 mM ALA and/or 50 ppm Cys significantly reduced the Chl a/b ratio compared to the untreated plants.

### 3.2. Oxidative Stress and Scavenging Capacity

**Figure 2:**
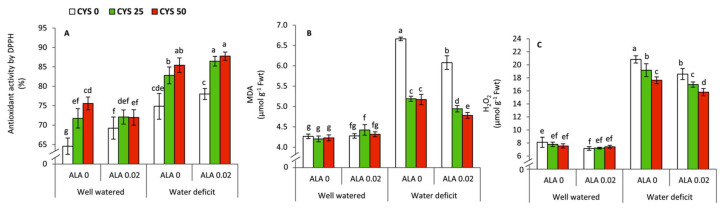
Effect of irrigation level, seed soaking by α-lipoic acid (ALA; 0 and 0.02 mM) and foliar application by cysteine (Cys; 0, 25 and 50 ppm) on the oxidative stress and scavenging capacity of wheat plants. (**A**) total antioxidant activity, (**B**) Lipid peroxidation (MDA), (**C**) Hydrogen peroxide content. For each parameter, the mean values ± SD followed by a different letter are significantly different according to Tukey’s range test (*p* ≤ 0.05).

To assess the efficiency of plants to scavenge the cytotoxic molecules which lead to the oxidative damage, antioxidant activity by DPPH, and lipid peroxidation as indicated by malondialdehyde (MDA) and H_2_O_2_ were determined (Table 3, Figure 2). The results showed that all above-mentioned variables were significantly affected by irrigation level (I), ALA as seed soaking (S) and Cys as foliar application (F) treatments. The interaction treatment of I × S was significant with H_2_O_2_, while the interaction treatment of I × F was significant with MDA and H_2_O_2_. The general tendency was that deficit irrigation resulted in a significant increase (*p* ≤ 0.05) in the antioxidant activity, MDA and H_2_O_2_ compared to the well watered plants. Despite being under water deficit, the treatment of 0.02 mM ALA individually reduced the concentration of MDA and H_2_O_2_, and the increase of antioxidant activity by DPPH did not reach the level of significance compared to ALA-untreated plants. The foliar applications of Cys (25/50 ppm) either individually or combined with ALA led to improve significantly (*p* ≤ 0.05) the antioxidant activity by DPPH in parallel with reducing the concentration of MDA and H_2_O_2_ under water deficit conditions.

### 3.3. Non-Enzymatic Antioxidants

**Figure 3:**
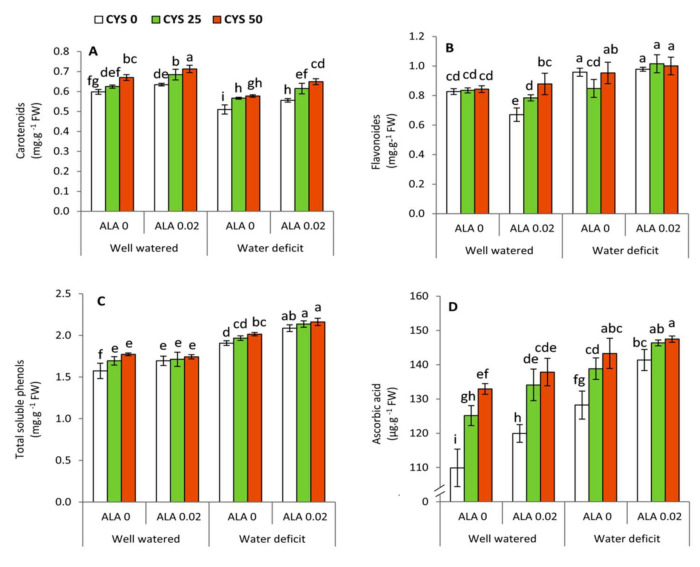
Effect of irrigation level, seed soaking by α-lipoic acid (ALA; 0 and 0.02 mM) and foliar application by cysteine (Cys; 0, 25 and 50 ppm) on the concentration of non-enzymatic antioxidants in wheat plants. (**A**) Carotenoids, (**B**) Flavonoids, (**C**) Total soluble phenols, (**D**) Ascorbic acid. For each parameter, the mean values ± SD followed by a different letter are significantly different according to Tukey’s range test (*p* ≤ 0.05).

Non-enzymatic antioxidants including carotenoids, total soluble phenols and ascorbic acid were significantly influenced by the irrigation level (I), ALA as seed soaking (S) and Cys as foliar application (F) treatments, whereas flavonoids were significantly affected by reducing the irrigation level (Table 4). The interactive treatments revealed that flavonoids and total soluble phenols were affected by I × S. Ascorbic acid was also significantly changed by I × F interaction treatment. Generally, carotenoids were significantly (*p* ≤ 0.05) decreased under water deficit. Conversely, flavonoids, total soluble phenols and ascorbic acid were increased with reducing the irrigation level (Figure 3). The highest significant (*p* ≤ 0.05) increase in carotenoids was obtained by the treatment of 0.02 mM ALA + 50 ppm Cys under well watered conditions. On the other hand, the treatment of 0.02 mM ALA + 50 ppm Cys achieved the maximum significant (*p* ≤ 0.05) increase in flavonoids, total soluble phenols and ascorbic acid compared to the untreated plants under water shortage.

### 3.4. Changes in the Activities of Antioxidant Enzymes

**Figure 4:**
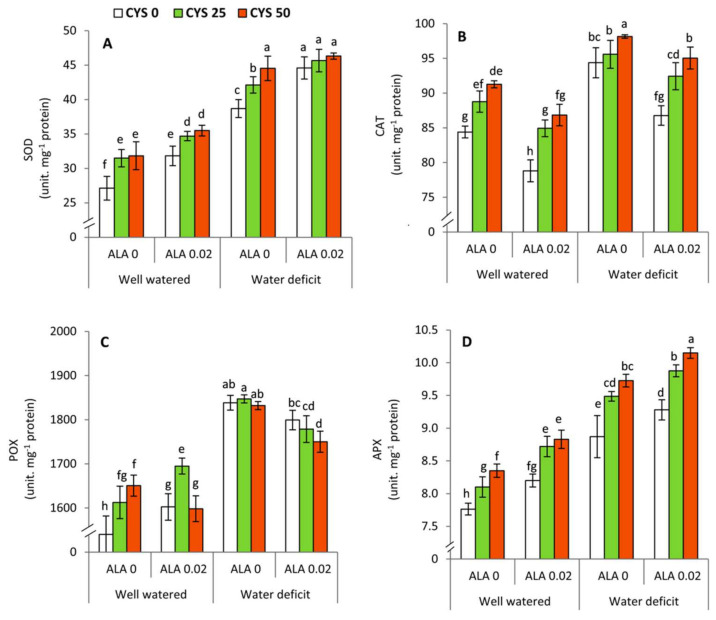
Effect of irrigation level, seed soaking by α-lipoic acid (ALA; 0 and 0.02 mM) and foliar application by cysteine (Cys; 0, 25 and 50 ppm) on the activities of antioxidant enzymes in wheat plants. (**A**) SOD, (**B**) CAT, (**C**) POX, (**D**) APX. For each parameter, the mean values ± SD followed by a different letter are significantly different according to Tukey’s range test (*p* ≤ 0.05).

The activities of antioxidant enzymes including SOD, CAT, POX, and APX were significantly affected by the irrigation level (I), ALA as seed soaking (S) and Cys as foliar application (F) treatments (Table 5). Moreover, POX was affected by all the interactions except the irrigation × seed soaking × Cys interaction. All antioxidant enzymes were increased under water deficit condition compared to well watered condition (Figure 4). Under water deficit conditions, SOD and APX were significantly (*p* ≤ 0.05) increased at higher ALA concentration (0.02 mM) compared to the untreated plants with ALA (0 mM). Conversely, the activity of CAT was significantly decreased at higher ALA concentration, whereas POX did not show any significant changes. All antioxidant enzymes were increased by increasing the concentrations of Cys except POX at higher ALA under well water condition. Under water shortage, the treatment of 50 ppm Cys without ALA and all Cys treatments with ALA recorded the maximum significant (*p* ≤ 0.05) activities in SOD. Additionally, the highest activity of CAT was achieved by the treatment of 50 ppm Cys without ALA under water deficit conditions, whereas the highest POX value was recorded at zero ALA concentration and 25 ppm Cys. Moreover, the treatment of 0.02 ALA + 50 ppm Cys achieved the highest significant (*p* ≤ 0.05) activity regarding to APX.

### 3.5. Changes in Osmolytes and Leaf Relative Water Content

**Figure 5:**
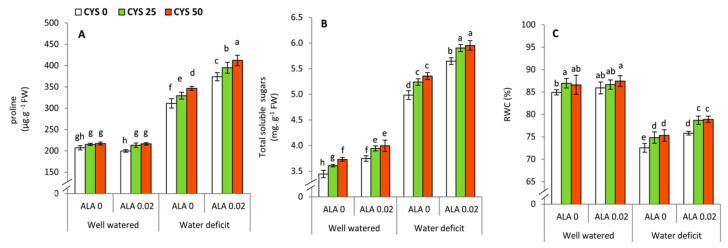
Effect of irrigation level, seed soaking by α-lipoic acid (ALA; 0 and 0.02 mM) and foliar application by cysteine (Cys; 0, 25 and 50 ppm) on the concentration of osmolytes and leaf relative water content (RWC) of wheat plants. (**A**) Proline, (**B**) Total soluble sugars, (**C**) RWC. For each parameter, the mean values ± SD followed by a different letter are significantly different according to Tukey’s range test (*p* ≤ 0.05).

Proline, total soluble sugars, and leaf relative water content (RWC) were significantly affected by the irrigation level (I), ALA as seed soaking (S) and Cys as foliar application (F) treatments (Table 6). The interaction treatments revealed that there was a significant relationship between the irrigation level and the seed soaking in ALA in respect to all traits (proline, total soluble sugars and RWC), as well as the interaction treatment of irrigation x foliar applications by Cys demonstrated a significant effect in proline. Generally, plants subjected to water deficit exhibited an obvious and significant (*p* ≤ 0.05) increase in both investigated osmolytes (proline and sugars). However, an opposite trend was observed in respect to RWC (Figure 5). The foliar applications of Cys at both examined concentrations (25/50 ppm) were showed to display a significant (*p* ≤ 0.05) increase in proline in ALA-treated plants under well watered conditions. In this respect, the treatment of 50 ppm Cys + 0.02 mM ALA recorded the highest significant values under water deficit conditions. In addition, total soluble sugars were significantly (*p* ≤ 0.05) improved by increasing the concentration of Cys either in ALA-treated or non-treated plants under both investigated levels of irrigation. Leaf relative water content (RWC) demonstrated remarkable and significant (*p* ≤ 0.05) improvement in the plants that exposed to both investigated concentrations of Cys under water deficit conditions.

### 3.6. Grain Yield and Its Components

**Figure 6:**
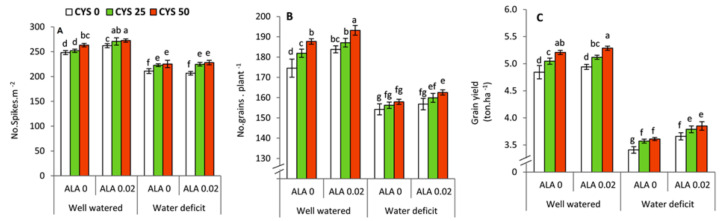
Effect of irrigation level, seed soaking by α-lipoic acid (ALA; 0 and 0.02 mM) and foliar application by cysteine (Cys; 0, 25 and 50 ppm) on grain yield and its components of wheat plants. (**A**) number of spikes, (**B**) number of grains, (**C**) Grain yield. For each parameter, the mean values ± SD followed by a different letter are significantly different according to Tukey’s range test (*p* ≤ 0.05).

Analysis of variance (Table 7) shows that number of spikes per m^2^, number of grains per plant and total grain yield (ton/ha) were significantly affected by the irrigation level (I), ALA as seed soaking (S) and Cys as foliar application (F) treatments. The interaction treatments demonstrated that number of spikes and total grain yield were affected by the interaction between the irrigation level and seed soaking treatment by ALA. Additionally, total grain yield was affected by the interaction between the irrigation level and Cys treatments. The highest significant (*p* ≤ 0.05) number of spikes/m^2^ was obtained by the treatment of ALA 0.02 + Cys 50 under well watered conditions (Figure 6A). Under limited water supply, plants that were treated by Cys (25 or 50 ppm) displayed a significant (*p* ≤ 0.05) increase in the number of spikes compared to the untreated plants regardless of the ALA treatments. Additionally, the highest number of grains per plant was achieved by the treatment of ALA 0.02 + Cys 50 under well watered conditions (Figure 6B). A similar trend was observed under water deficit conditions. Enhancement of the number of spikes and number of grains by ALA and Cys treatments was eventually reflected on the total grain yield (ton/ha) (Figure 6C).

### 3.7. The Physical/Chemical Properties of Grains

Quality assessment of wheat grains was performed by evaluating some simple physical/chemical parameters (Table 8). All studied parameters including moisture content, wet gluten, gluten index, falling number, weight of 1000 kernels and hectoliter weight were significantly affected by reducing the irrigation level. Moreover, ALA as a seed soaking treatment significantly affected gluten index, whereas applied Cys significantly affected moisture content, gluten index, falling number and weight of 1000 kernels. No significant effects were detected between all possible interaction treatments except irrigation x Cys treatments in respect to the moisture content and falling number.

**Figure 7:**
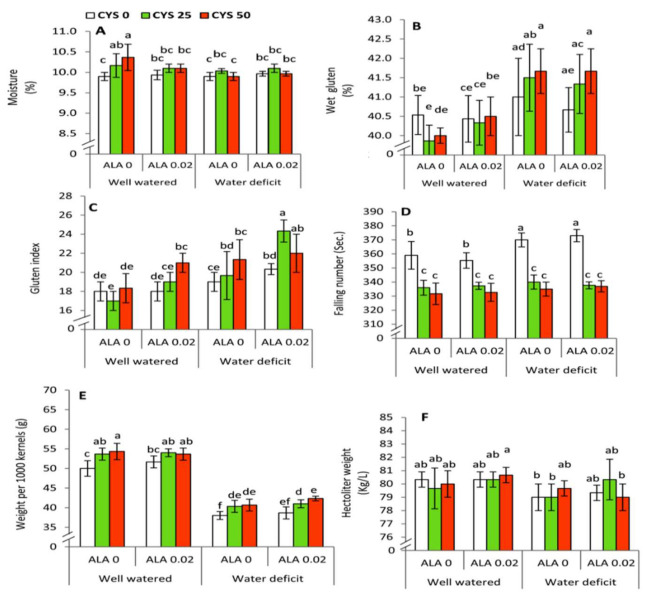
Effect of irrigation level, seed soaking by α-lipoic acid (ALA; 0 and 0.02 mM) and foliar application by cysteine (Cys; 0, 25 and 50 ppm) on the physical/chemical properties of wheat grains. (**A**) Moisture content, (**B**) Wet gluten, (**C**) Gluten index, (**D**) Falling number, (**E**) Weight per 1000 kernels, (**F**) Hectoliter weight. For each parameter, the mean values ± SD followed by a different letter are significantly different according to Tukey’s range test (*p* ≤ 0.05).

The highest significant (*p* ≤ 0.05) moisture content (Figure 7A) was obtained by the treatment of 50 ppm Cys under well watered conditions compared to the control. The wet gluten (Figure 7B) revealed an obvious and significant (*p* ≤ 0.05) increase under water deficit conditions especially with increasing the concentration of Cys up to 50 ppm. The highest significant (*p* ≤ 0.05) value in the gluten index (Figure 7C) was achieved by the treatment of 0.02 mM ALA + 25 ppm Cys under deficit irrigation conditions. Falling number displayed a significant (*p* ≤ 0.05) decrease in all treatments of Cys at both examined concentrations compared to the Cys-untreated plants (Figure 7D). This increase was more pronounced under deficit irrigation compared to the well watered conditions. The weight of 1000 kernels (Figure 7E) was significantly (*p* ≤ 0.05) increased in parallel with rising the concentration of Cys up to 50 ppm under both investigated levels of irrigation, but the treatment of 0.02 mM ALA did not show any significant results. Furthermore, no significant changes were observed in the hectoliter weight by all investigated treatments under the same level of irrigation (Figure 7F). However, the treatment of 0.0 mM ALA + 50 ppm Cys under well watered condition revealed a significant increase compared to the treatments of zero ALA + zero Cys, zero ALA + 25 ppm Cys and 0.02 mM ALA + 50 ppm Cys under water limited supply.

### 3.8. Alveographic Parameters of Wheat Grains

**Figure 8:**
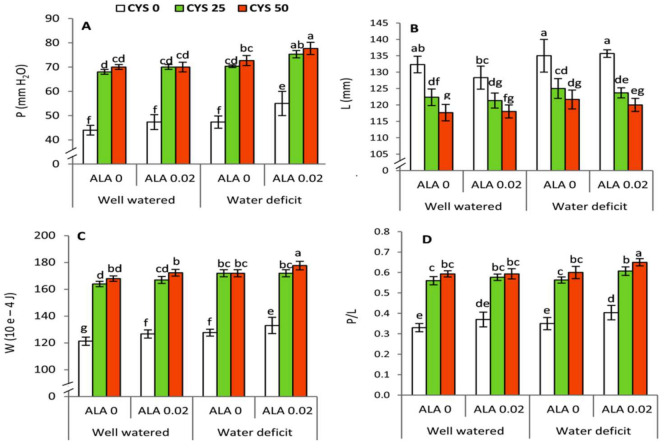
Effect of irrigation level, seed soaking by α-lipoic acid (ALA; 0 and 0.02 mM) and foliar application by cysteine (Cys; 0, 25 and 50 ppm) on the alveographic parameters of wheat grains. (**A**) P, (**B**) L, (**C**) W, (**D**) P/L. For each parameter, the mean values ± SD followed by a different letter are significantly different according to Tukey’s range test (*p* ≤ 0.05).

All alveographic parameters of wheat grains were significantly affected by irrigation, seed soaking, and foliar application treatments interaction except seed soaking of *L* (Table 9, Figure 8). All alveographic parameters of wheat grains were significantly increased by water deficit, high concentration of ALA (except *L*), and the highest Cys concentration (except *L*). *L* values were decreased with increasing Cys concentration.

### 3.9. Correlation between Irrigation, Seed Soaking and Foliar Application Parameters

**Figure 9:**
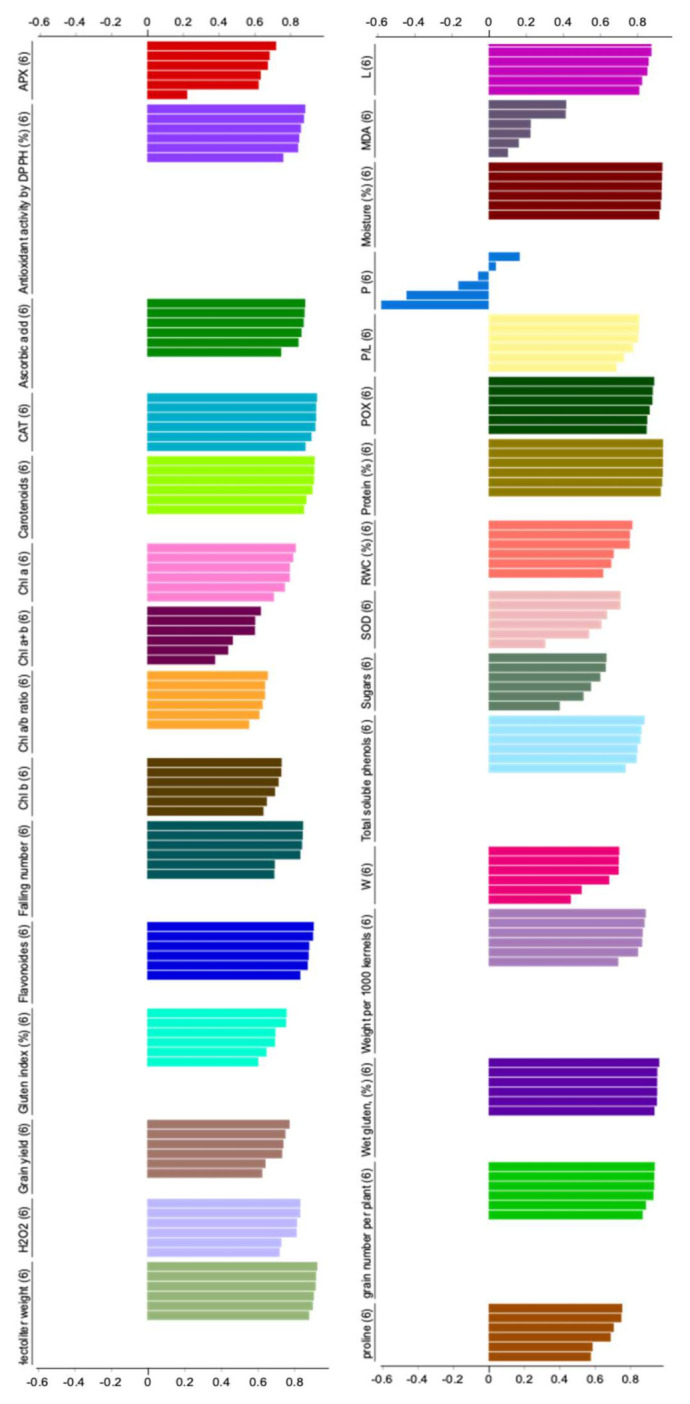
Plot of silhouette analysis values for clustering of all parameters based on “seed soaking and foliar application” treatments variables. On the y-axis each cluster is ordered by decreasing silhouette value. The silhouette value can range between −1 and 1.

**Figure 10:**
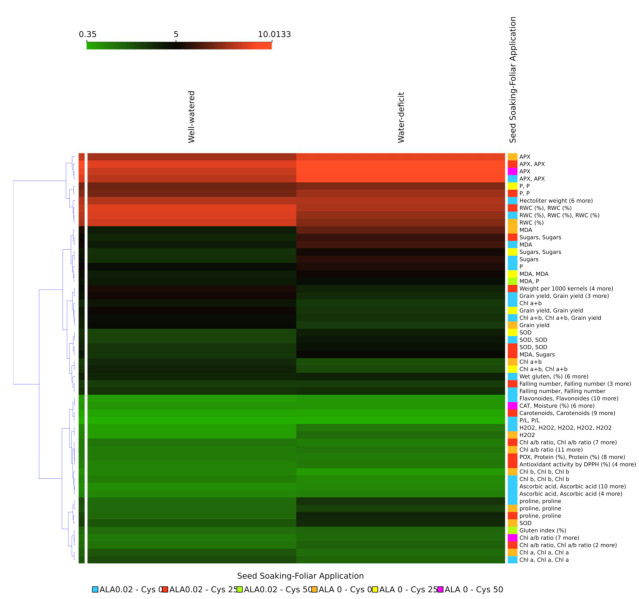
Two-dimensional heatmap visualization shows the interaction between the irrigation treatments and both the 31 measured parameters included in the study and the six “seed soaking and foliar application” types.

To assess the cluster quality of treatments based on the irrigation, seed soaking, and foliar application treatments measurements by testing the cluster distances within and between each cluster, the silhouette analysis plot was calculated and generated based on the Euclidean distance metric (Figure 9). The results revealed that all the parameters except P exhibited positive values indicating that clustering of treatments based on the irrigation and seed soaking and foliar application showed mostly similar behavior with different levels of effect, and the clustering configuration may have few clusters. Meanwhile, two-dimensional heatmap plotting based on all parameters clustered the APX, P, and RWC (%) in a separate cluster, indicating that these parameters almost have the similar power to elucidate the water deficit effects when compared to the well watered condition. While the second cluster comprised two distinctive sub-clusters, the first comprised MDA, sugars, grain yield, weight per 1000 kernels, SOD, Chl a+b, wet gluten (%), and falling number parameters/traits. At the same time, the second sub-cluster comprised Chl a, Chl a/b, proline, ascorbic acid, Chl b, protein (%), POX, H_2_O_2_, CAT, carotenoids, moisture (%), P/L, and flavonoids parameters/traits (Figure 10). Boxplot analysis revealed that the average of the parameter-classes leaf pigments, grain yield, and physical/chemical properties of wheat grain classes decreased significantly under the water deficit compared to well watered condition. The classes of non-enzymatic antioxidants, ROS and scavenging capacity, antioxidant enzymes, osmolytes, leaf water status, and alveographic parameters of wheat grains increased the water deficit significantly compared to well watered condition (Supplementary Table S1).

**Figure 11:**
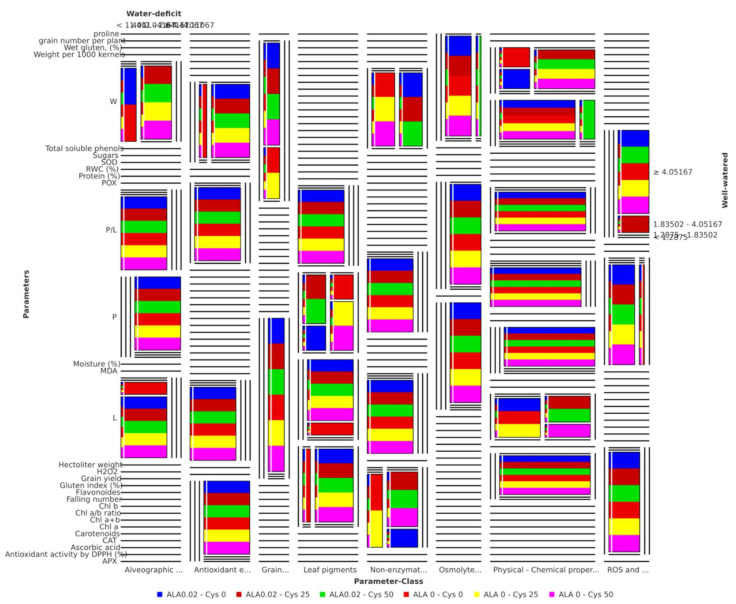
Mosaic plot representing a contingency table of the water deficit and well watered treatments variants versus the 31 parameters included in the study. The vertical size of the cells is proportional to the number of variants found in the respective parameter; the horizontal size of the cells is proportional to the effect level of the six “seed soaking and foliar application” types for each parameter under each parameter-class. The colors of the variants (seed soaking and foliar application) are indicated at the bottom of the Mosaic plot in colored squares similar to the respective bars in the Mosaic plot. Variants were not found at all possible locations of each parameter-class, which causes the reduction of several bars to dashed lines drawn as place holders and indicating that at the particular location no variant has been found in the parameter-class.

Meanwhile, Boxplot representation of the “seed soaking and foliar application” treatments on both water deficit and well watered conditions revealed that the average of all treatments except treatments ALA 0.02 - Cys 25 and ALA 0.02 - Cys 50 showed minor variations under the water deficit compared well watered condition (Supplementary Table S1). Moreover, multidimensional preference analysis was performed to summarize the interrelationships amongst treatments, parameters, and classes. The plot shows a high level of consistency and interrelationships between each parameter and treatment type. Ultimately, to summarize and visualize such high-dimensional data levels, a Mosaic plot representing a contingency matrix of the water deficit and well watered treatments versus the 31 parameters and their classes included in the study was developed (Figure 11). The plot further confirmed that the six “seed soaking and foliar application” treatments applied on both water deficit and well watered plant groups exhibited different levels of effectiveness (moderate to minor) to alleviate the negative effect of water deficit when compared to well watered condition.

## 4. Discussion

In the present study, exposing wheat plants to deficit irrigation manifestly altered the leaf chlorophyll composition. In this respect, it can be observed that Chl a, Chl b, Chl a+b contents were significantly decreased in the water stressed plants compared to the well watered ones (Figure 1A–C). This reduction could be attributed to increase the activity of chlorophyllase [62], as well as water deficiency induced oxidative damage (Figure 2B,C) which can collapse the membranes and chloroplast structure where the leaf pigments are localized [63,64]. In contrast, the Chl a/b ratio (Figure 1D) in ALA/Cys-untreated plants was significantly increased under water deficit conditions compared to the well watered plants. It is well known that Chl a is the major cofactor for the photochemical reactions in the plastid because it is required for the assembly of pigment–protein complexes, while Chl b can also act as one of the accessory pigments in light-harvesting chlorophyll complexes (LHCs) [65,66]. In this study, increasing the Chl a/b ratio may act as a protective and adaptive behavior to maintain the function of photosynthetic apparatus under water stress conditions.

On the other hand, applied ALA and/or Cys were shown to enhance the content of photosynthetic pigments (Chl a, Chl b and Chl a+b) under well watered and water stressed conditions. However, an opposite trend was observed in Chl a/b ratio (Figure 1). Sulfur-containing biomolecules (ALA and Cys) are considered very potent antioxidants that can protect plants against abiotic stresses [24,30]. In this context, exogenous ALA was shown to stimulate photosystem II and gene expression of carbon fixation enzymes (Rubisco and PEP carboxylase) in maize seedlings exposed to drought stress [29]. These responses were associated with a simultaneous down-regulation in the chlorophyllase gene (Chlase) [29]. Moreover, Cys has been reported to improve Chl a and Chl b content in oat plants subjected to drought stress [67]. This effect could be attributed to enhancing the uptake of N in the plants, which is essential for Chl biosynthesis [68]. In contrast, the Chl a/b ratio (Figure 1D) differentially responded to ALA treatments under water deficit conditions. It was significantly decreased in the ALA-treated plants compared to the ALA-untreated plants. This effect implies that ALA as an efficient antioxidant has a more positive impact on Chl b than Chl a.

In the present study, water shortage led to an increase of H_2_O_2_ and the rate of lipid peroxidation as indicated by malondialdehyde (MDA) (Figure 2B,C). The excessive generation of reactive oxygen species (ROS) under drought stress conditions has been evidenced in several plant species [69,70,71,72]. This increase of toxic molecules should be restricted by enhancing the overall antioxidant activities and scavenging capacity of plants (Figure 2A). In this context, the treatments of ALA/Cys revealed beneficial effects as protectant antioxidants by reducing MDA and H_2_O_2_ in parallel with enhancing the antioxidant capacity by DPPH (Figure 2A–C). Previous investigations on the antioxidative role of ALA during Pb toxicity revealed ALA-induced accumulation of various antioxidant molecules in wheat plants [73]. Furthermore, ALA can enhance the photosynthetic performance in maize under drought stress [29], ameliorate lipid peroxidation, and induce the antioxidant systems of maize under osmotic stress [25]. Cysteine (Cys) has also been suggested as an important precursor for the synthesis of glutathione in plants [24].

Carotenoids were significantly decreased in the water stressed plants compared to the well watered plants (Figure 3A). This response could be explained by that carotenoids are involved in the biosynthesis of ABA, the major signaling hormone in response to drought stress [74]. In contrast, the treatments of ALA/Cys improved the content of carotenoids under deficit irrigation. This synergistic effect may have occurred to keep the carotenoids that mediate the xanthophyll cycle, leading to dissipating the exceeding light energy and enhancing the photosynthetic capacity under drought stress. In this regard, earlier evidence has confirmed that Cys can hinder energy transfer to prevent photooxidation [75].

On the other hand, flavonoids and total soluble phenols revealed a significant increase in the water stressed plants compared to the well watered ones (Figure 3B,C). This over-accumulation of secondary metabolites can enhance plant tolerance to drought stress and alleviate the induced oxidative damage due to their higher antioxidant capacity [76,77]. In addition to their antioxidant properties, phenolic compounds can be involved in plant tolerance to drought stress as a sink for carbon under stress conditions [78]. Furthermore, flavonoids are widely distributed secondary metabolites that are synthesized through the phenylpropanoid pathway, transforming phenylalanine into 4-coumaroyl-CoA, which finally enters the flavonoid biosynthesis pathway [79]. The carbon skeleton of all secondary metabolites including the compound-mediated phenylpropanoid pathway basically relies on photosynthesis, since several changes in the stressed plants happen in carbon metabolism to achieve the balance between the biomass production and formation of defensive secondary compounds [76]. In this study, flavonoids and total soluble phenols were relatively enhanced by the combined ALA/Cys treatments. This enhancement could be attributed to the increase of the rate of photosynthesis by both compounds [29,80].

Alpha lipoic acid (ALA) is soluble in water and lipid phases, connects the activity of antioxidants in the cell membrane (α-tocopherol) with other antioxidants in the cytoplasm, i.e., ascorbic acid (AsA) and glutathione (GSH), leading to reinforcing the antioxidant power of plants [31]. Moreover, Cys is an important precursor of GSH biosynthesis in plants [24]. In the current work, the treatments of ALA/Cys increased the concentration of AsA under both investigated levels of irrigation (Figure 3D). These findings indicate that the treatments of ALA/Cys can play a modulatory role in the operation of the GSH-AsA cycle under the circumstances of this study [81].

The activities of antioxidant enzymes including superoxide dismutase (SOD), catalase (CAT), peroxidase (POX) and ascorbate peroxidase (APX) were observed to be significantly affected by deficit irrigation and ALA/Cys treatments (Table 5; Figure 4). Interestingly, ALA and Cys exhibited differential effects on the activity of antioxidative enzymes, whereas SOD and APX activity were positively upregulated by ALA treatment while CAT and POX activity were decreased. Thus, ALA functions through the activation of the major ROS scavenging system that includes APX and SOD. Reduction of H_2_O_2_ content was accompanied by ALA and Cys-mediated elevation in APX, POX, and CAT activity. Further investigations are necessary to reveal the mechanism of ALA and Cys signaling in the modulation of antioxidative enzymes in specific organelles in response to water stress in wheat leaves. Interestingly, Cys treatment during water deficit elevated the activity of all antioxidative enzymes, which was all the higher for POX. Thus, the present work highlights the integrative and synergistic role of ALA and Cys application in the upregulation of the enzymatic antioxidative defense systems. It is further advocated that both ALA and Cys are beneficial to instigate redox management and ROS scavenging activity [25,30,67,73]. Exogenously applied ALA and Cys have been suggested to mitigate the oxidative stress and confer osmotic tolerance to wheat and soybean plants subjected to NaCl stress [30,68].

In the present work, ALA and Cys treatments also increased the proline, total soluble sugars and leaf relative water content (RWC) of drought stressed wheat plants (Table 6, Figure 5). Several lines of evidence demonstrated that to regulate the osmotic potential and enhance water uptake under osmotic stress (drought or salinity), plants usually accumulate considerable concentrations of some organic molecules such as proline and sugars that function as osmotic regulators and enhance plant water relation [64,82,83].

It is noteworthy that the improved osmotic regulation in leaves was also associated with the amelioration of yield attributes (Table 7, Figure 6) including number of spikes/m^2^, number of grains/plant and grain yield (ton/ha). Several previous studies reported that water deficit has a destructive effect on the yield of wheat plants [84,85]. Generally, water stress can affect many aspects related to the field performance of grain crops such as the photosynthesis and translocation of carbohydrates, which are responsible for the filling of grains [86,87]. In this study, besides the amelioration of drought induced oxidative damage, the treatments of ALA/Cys were evidenced to enhance photosynthetic pigments, accumulation of osmolytes and improving the non-enzymatic and enzymatic antioxidants. These positive effects could explain the increase of grain yield attributes.

ALA and Cys treatments improved moisture percentage and gluten index in grains obtained from water stressed plants. Cys treatment further improved kernel weight (per 1000 kernels) under water deficit conditions. Analysis of alveographic characteristics indicated that ALA and Cys applications were effective in improving flour and dough quality of wheat grains. Total amino acid content, water relation, and growth characteristics are known to be associated with physiochemical properties, as well as grain quality in drought stressed wheat plants [88].

Additionally, Cys and ALA treatments (sulfur-containing molecules) significantly improved grain attributes. In recent years, investigations have revealed the emerging role of ALA (dithiol) as a potential antioxidant in plants [89]. Reduced ALA possesses 2 sulfhydryl moieties which function as potential ROS scavenging sites. ALA is an important sulfur-bearing compound that exerts a plethora of effects in plants subjected to abiotic stress [73]. ALA further activates a number of mitochondria-localized metabolic enzymes [90]. Sulfur compounds are important regulators of signaling pathways associated with abiotic stress tolerance in plants [91,92]. In the present work, application of the two sulfur-containing priming molecules (ALA and Cys) led to several synergistic effects that enhanced the tolerance of wheat plants to water shortage.

Various physiochemical attributes have been improved in the presence of ALA and Cys treatment during water stress in wheat plants. Future investigations are necessary to establish the possible role of ALA and Cys in the modulation of the glutathione-ascorbate cycle in water stressed wheat plants. It is noteworthy that application of the two biomolecules has led to an improvement in the gluten index and quality of grains (Table 8; Figure 7). Thus, using ALA and Cys are effective in the grain filling stage of wheat plants under water stress. This effect may indicate the presence of long distance signaling of ALA and Cys from leaves to grains. Correlation analysis revealed significant interaction among irrigation, seed soaking, and ALA/Cys treatments in wheat plants raised in the absence and presence of drought stress. Thus, ALA and Cys are effective priming molecules and bear agronomic importance in improving wheat yield in the arid cultivable regions. Priming for drought or salinity allows stress tolerance and offers several benefits in many crops [93]. This improvement was extended to the grain processing technology by affecting the alveographic parameters (Table 8 and Table 9). Analysis of alveographic characteristics indicated that ALA and Cys application were effective in improving flour and dough quality of wheat grains. In our present work, Cys and ALA treatment (sulfur-containing molecules) significantly improved grain attributes. Various physiochemical attributes of grains have improved in the presence of ALA and Cys treatment during water stress in wheat plants, and alveographic parameters of wheat flour dough also was improved. This improvement extended to the grain processing technology by affecting the alveographic parameters (technological quality parameters). Such parameters are strongly related to the flour yield and flour properties by enhancing the viscoelastic properties of dough [94]. In this context, it has been found that altering water relation and total amino acid content is concomitant with substantial changes in the physiochemical properties and grain quality of drought stressed wheat plants [88]. 

## 5. Conclusions

The present work provides novel insights into the synergistic action of ALA and Cys as effective stress priming molecules in the mitigation of drought stress in wheat plants. Amelioration of drought stress in wheat plants is primarily attained by the enhancement in the function of the antioxidative system and regeneration of osmotic tolerance in leaves. Apart from their antioxidative role, these sulfur-containing compounds also appear beneficial in the improvement of alveographic characteristics of wheat grain which determine various attributes of dough quality. Therefore, applications of these two biomolecules provide both physiological tolerance and restore yield attributes in wheat. Future investigations are necessary to decipher the signaling mechanisms of these two biomolecules in relation to various plant growth regulators in wheat plants. The molecular mechanism of ALA-Cys crosstalk shall also provide new insights to wheat management in arid regions. From our obtained results it can be concluded that applied ALA at 0.02 mM as seed soaking treatment combined by Cys at 50 ppm as a foliar application could be recommended as potent compounds in wheat cultivation under water deficit conditions.

## Figures and Tables

**Table 1:** Details of irrigation requirements of two consecutive seasons (2018/2019 and 2019/2020) for wheat based on the reference evapotranspiration calculated by FAO 56 Penman–Monteith equation. The irrigation system efficiency of 75% is considered in the calculations.

Growth Stage	Kc	Season			
		2018/2019		2019/2020	
		Mean ET_o_, mm	Total IR, m^3^/ha	Mean ET_o_, mm	Total IR, m^3^/ha
Initial	0.7	3.1	578.7	3.9	728.0
Crop development	1.15	2.8	2037.4	2.8	2025.6
Mid season	1.15	3.8	4081.7	3.4	3687.7
Late season	0.25	5.9	1105.0	5.3	984.9
Total IR, m^3^/ha			7802.8		7426.2

**Table 2:** Analysis of variance (mean square) of leaf chlorophyll concentration.

Source	Df	Chl a	Chl b	Chl a+b	Chl a/b
		(mg·g^−1^ FW)	(mg·g^−1^ FW)	(mg·g^−1^ FW)	Ratio
Irrigation (I)	1	3.561 ***	1.746 ***	10.304 ***	0.399 ***
Seed soaking (S)	1	0.669 ***	0.783 ***	2.901 ***	0.708 ***
Foliar appl. (F)	2	0.067 ***	0.033 ***	0.196 ***	0.010 **
I × S	2	0.031 ***	0.101ns	0.019 **	0.664 ***
I × F	2	0.004ns	0.001ns	0.003ns	0.014 **
S × F	2	0.001ns	0.001ns	0.000ns	0.011 **
I × S × F	2	0.001ns	0.002ns	0.000ns	0.000ns

ns, **, *** not significant or significant at *p* ≤ 0.01, *p* ≤ 0.001, analysis of variance.

**Table 3:** Analysis of variance (mean square) of ROS and scavenging capacity.

Source	Df	Antioxidant Activity	MDA	H_2_O_2_
		by DPPH (%)	(µmol g^−1^ FW)	(µmol g^−1^ FW)
Irrigation (I)	1	1229.671 ***	15.484 ***	1015.590 ***
Seed soaking (S)	1	27.737 ***	0.697 *	15.906 ***
Foliar appl. (F)	2	239.534 ***	2.350 ***	7.408 ***
I × S	2	15.210ns	0.148ns	5.313 ***
I × F	2	10.787ns	1.528 ***	6.077 ***
S × F	2	15.547ns	0.045ns	0.314ns
I × S × F	2	10.570ns	0.176ns	0.036ns

ns, *, *** not significant or significant at *p* ≤ 0.05, *p* ≤ 0.001, analysis of variance.

**Table 4:** Analysis of variance (mean square) of non-enzymatic antioxidants.

Source	Df	Carotenoids	Flavonoids	Total Soluble Phenols	Ascorbic Acid
		(mg·g ^−1^ FW)	(mg·g^−1^ FW)	(mg·g^−1^ FW)	(µg·g^−1^ FW)
Irrigation (I)	1	0.055 ***	0.225 ***	1.046 ***	1836.1225 ***
Seed soaking (S)	1	0.026 ***	0.000ns	0.101 ***	592.922 ***
Foliar appl. (F)	2	0.021 ***	0.009ns	0.034 ***	771.960 ***
I × S	2	0.001ns	0.026 *	0.043 ***	0.233ns
I × F	2	0.001ns	0.006ns	0.001ns	77.160 **
S × F	2	0.001ns	0.008ns	0.006ns	37.307ns
I × S × F	2	0.001ns	0.005ns	0.004ns	4.423ns

ns, *, **, *** not significant or significant at *p* ≤ 0.05, *p* ≤ 0.01, *p* ≤ 0.001, analysis of variance.

**Table 5:** Analysis of variance (mean square) of antioxidant enzymes.

Source	Df	SOD	CAT	POX	APX
		(unit. mg^−1^ protein)	(unit. mg^−1^ protein)	(unit. mg^−1^ protein)	(unit. mg^−1^ protein)
Irrigation (I)	1	1207.313 ***	560.742 ***	32852.022 ***	13.788 ***
Seed soaking (S)	1	129.925 ***	191.730 ***	2352.249 *	1.904 ***
Foliar appl. (F)	2	51.214 ***	140.043 ***	4573.021 **	1.713 ***
I × S	2	0.0270ns	1.343ns	19834.022 ***	0.024ns
I × F	2	1.470ns	2.729ns	7253.027 ***	0.051ns
S × F	2	5.391ns	8.817ns	5868.582 **	0.005ns
I × S × F	2	1.894ns	2.335ns	2776.361ns	0.009ns

ns, *, **, *** not significant or significant at *p* ≤ 0.05, *p* ≤ 0.01, *p* ≤ 0.001, analysis of variance.

**Table 6:** Analysis of variance (mean square) of osmolytes and leaf water status.

Source	Df	Proline	Sugars	Leaf RELATIVE Water Content (RWC)
		(µg·g^−1^ FW)	(mg·g^−1^ FW)	(%)
Irrigation (I)	1	202155.146 ***	28.178 ***	973.856 ***
Seed soaking (S)	1	8405.833 ***	2.002 ***	37.576 ***
Foliar appl. (F)	2	1919.463 ***	0.295 ***	18.286 ***
I × S	2	10332.723 ***	0.258 ***	21.098 **
I × F	2	414.193 ***	0.005ns	1.639ns
S × F	2	21.095ns	0.003ns	0.157ns
I × S × F	2	2.801ns	0.006ns	0.735ns

ns, **, *** not significant or significant at *p* ≤ 0.01, *p* ≤ 0.001, analysis of variance.

**Table 7:** Analysis of variance (mean square) of grain yield and its components.

Source	Df	Spike Number	Grain Number	Grain Yield
		per m^2^	per plant	(ton·ha^−1^)
Irrigation (I)	1	15604.17 ***	6445.413 ***	18.261 ***
Seed soaking (S)	1	447.32 ***	238.702 ***	0.224 ***
Foliar appl. (F)	2	733.24 ***	190.810 ***	0.230 ***
I × S	2	433.33 ***	20.400ns	0.054 **
I × F	2	60.02ns	32.418ns	0.021 *
S × F	2	24.92ns	1.740ns	0.001ns
I × S × F	2	30.95ns	7.463ns	0.002ns

ns, *, **, *** not significant or significant at *p* ≤ 0.05, *p* ≤ 0.01, *p* ≤ 0.001, analysis of variance.

**Table 8:** Analysis of variance (mean square) of physical and chemical properties of grains.

Source	Df	Moisture	Wet Gluten	Gluten Index	Falling Number	Weight per 1000	Hectoliter Weight
		(%)	(%)	(%)	Sec.	kernels (g)	kg/L
Irrigation (I)	1	0.122 *	9.506 ***	58.778 ***	413.444 **	1534.027 ***	6.250 *
Seed soaking (S)	1	0.003ns	0.034ns	32.111 **	0.444ns	0.251ns	1.361ns
Foliar appl. (F)	2	0.112 *	0.280ns	10.333 *	3270.361 ***	11.861 *	0.027ns
I × S	2	0.063ns	0.466ns	1.000ns	4.000ns	0.694ns	0.027ns
I × F	2	0.081 *	1.041ns	4.777ns	130.527 *	0.027ns	0.583ns
S × F	2	0.017ns	0.181ns	5.444ns	3.694ns	1.750ns	0.861ns
I × S × F	2	0.017ns	0.031ns	4.333ns	20.083ns	0.361ns	0.861ns

ns, *, **, *** not significant or significant at *p* ≤ 0.05, *p* ≤ 0.01, *p* ≤ 0.001, analysis of variance.

**Table 9:** Analysis of variance (mean square) of alveographic parameters of wheat grains.

Source	Df	P	L	W	P/L
		(mm H_2_O)	(mm)	(10^−4^ J)	
Irrigation (I)	1	210.254 ***	110.250 ***	306.250 ***	0.005 **
Seed soaking (S)	1	132.255 ***	12.250ns	140.027 ***	0.010 ***
Foliar appl. (F)	2	2186.111 ***	582.750 ***	7596.694 ***	0.214 ***
I × S	2	38.022ns	1.361ns	0.694ns	0.002ns
I × F	2	2.333ns	5.250ns	3.083ns	0.001ns
S × F	2	7.001ns	0.750ns	13.527ns	0.001ns
I × S × F	2	0.777ns	9.027ns	3.694ns	0.001ns

ns, **, *** not significant or significant at *p* ≤ 0.01, *p* ≤ 0.001, analysis of variance.

## Data Availability

Not applicable.

## Supplementary Materials

The following are available online at https://www.mdpi.com/article/10.3390/plants10112318/s1, Table S1: Germination assay of Wheat seed after soaking with different concentration of α-lipoic acid.

## References

[1] Elkeilsh A., Awad Y.M., Soliman M.H., Abu-Elsaoud A., Abdelhamid M.T., El-Metwally I.M. (2019). Exogenous application of β-sitosterol mediated growth and yield improvement in water-stressed wheat (*Triticum aestivum*) involves up-regulated antioxidant system. J. Plant Res..

[2] Sallam A., Alqudah A.M., Dawood M.F., Baenziger P.S., Börner A. (2019). Drought stress tolerance in wheat and barley: Advances in physiology, breeding and genetics research. Int. J. Mol. Sci..

[3] Sharar M., Saied E.M., Rodriguez M.C., Arenz C., Montes-Bayón M., Linscheid M.W. (2017). Elemental Labelling and Mass Spectrometry for the Specific Detection of Sulfenic Acid Groups in Model Peptides: A Proof of Concept. Anal Bioanal Chem.

[4] Lal R. (2004). Soil carbon sequestration impacts on global climate change and food security. Science.

[5] Ahmad P., Jamsheed S., Hameed A., Rasool S., Sharma I., Azooz M., Hasanuzzaman M. (2014). Drought stress induced oxidative damage and antioxidants in plants. Oxidative Damage to Plants.

[6] Alhaithloul H.A., Soliman M.H., Ameta K.L., El-Esawi M.A., Elkelish A. (2020). Changes in Ecophysiology, Osmolytes, and Secondary Metabolites of the Medicinal Plants of Mentha piperita and Catharanthus roseus Subjected to Drought and Heat Stress. Biomolecules.

[7] Habib N., Ali Q., Ali S., Javed M.T., Zulqurnain Haider M., Perveen R., Shahid M.R., Rizwan M., Abdel-Daim M.M., Elkelish A. (2020). Use of Nitric Oxide and Hydrogen Peroxide for Better Yield of Wheat (*Triticum aestivum* L.) under Water Deficit Conditions: Growth, Osmoregulation, and Antioxidative Defense Mechanism. Plants.

[8] Harb A., Krishnan A., Ambavaram M.M., Pereira A. (2010). Molecular and physiological analysis of drought stress in Arabidopsis reveals early responses leading to acclimation in plant growth. Plant Physiol..

[9] Gaber A., Refat M.S., Belal A.A.M., El-Deen I.M., Hassan N., Zakaria R., Alhomrani M., Alamri A.S., Alsanie W.F., Saied E.M. (2021). New Mononuclear and Binuclear Cu(II), Co(II), Ni(II), and Zn(II) Thiosemicarbazone Complexes with Potential Biological Activity: Antimicrobial and Molecular Docking Study. Molecules.

[10] Miller G., Suzuki N., Ciftci-Yilmaz S., Mittler R. (2010). Reactive oxygen species homeostasis and signalling during drought and salinity stresses. Plant Cell Environ..

[11] Noctor G., Mhamdi A., Foyer C.H. (2014). The roles of reactive oxygen metabolism in drought: Not so cut and dried. Plant Physiol..

[12] Elkelish A., Ibrahim M.F., Ashour H., Bondok A., Mukherjee S., Aftab T., Hikal M., El-Yazied A.A., Azab E., Gobouri A.A. (2021). Exogenous Application of Nitric Oxide Mitigates Water Stress and Reduces Natural Viral Disease Incidence of Tomato Plants Subjected to Deficit Irrigation. Agronomy.

[13] Ibrahim M.F., Elbar O.H.A., Farag R., Hikal M., El-Kelish A., El-Yazied A.A., Alkahtani J., Abd El-Gawad H.G. (2020). Melatonin counteracts drought induced oxidative damage and stimulates growth, productivity and fruit quality properties of tomato plants. Plants.

[14] Bin-Jumah M., Abdel-Fattah A.-F.M., Saied E.M., El-Seedi H.R., Abdel-Daim M.M. (2021). Acrylamide-Induced Peripheral Neuropathy: Manifestations, Mechanisms, and Potential Treatment Modalities. Environ. Sci. Pollut. Res..

[15] Nawaz F., Shehzad M.A., Majeed S., Ahmad K.S., Aqib M., Usmani M.M., Shabbir R.N. (2020). Role of Mineral Nutrition in Improving Drought and Salinity Tolerance in Field Crops. Agronomic Crops.

[16] Gaber A., Alsanie W.F., Kumar D.N., Refat M.S., Saied E.M. (2020). Novel Papaverine Metal Complexes with Potential Anticancer Activities. Molecules.

[17] Huang G.-T., Ma S.-L., Bai L.-P., Zhang L., Ma H., Jia P., Liu J., Zhong M., Guo Z.-F. (2012). Signal transduction during cold, salt, and drought stresses in plants. Mol. Biol. Rep..

[18] Ibrahim M., Ibrahim H.A. (2016). Assessment of Selenium Role in Promoting or Inhibiting Potato Plants under Water Stress. J. Hortic. Sci. Ornam. Plants.

[19] Ibrahim M.F., El-Samad A., Ashour H., El-Sawy A.M., Hikal M., Elkelish A., El-Gawad H.A., El-Yazied A.A., Hozzein W.N., Farag R. (2020). Regulation of agronomic traits, nutrient uptake, osmolytes and antioxidants of maize as influenced by exogenous potassium silicate under deficit irrigation and semiarid conditions. Agronomy.

[20] Reddy A.R., Chaitanya K.V., Vivekanandan M. (2004). Drought-induced responses of photosynthesis and antioxidant metabolism in higher plants. J. Plant Physiol..

[21] Zou J.-J., Wei F.-J., Wang C., Wu J.-J., Ratnasekera D., Liu W.-X., Wu W.-H. (2010). Arabidopsis calcium-dependent protein kinase CPK10 functions in abscisic acid-and Ca^2+^-mediated stomatal regulation in response to drought stress. Plant Physiol..

[22] Colville L., Kranner I. (2010). Desiccation tolerant plants as model systems to study redox regulation of protein thiols. Plant Growth Regul..

[23] Meyer A.J., Hell R. (2005). Glutathione homeostasis and redox-regulation by sulfhydryl groups. Photosynth. Res..

[24] Zagorchev L., Seal C.E., Kranner I., Odjakova M. (2013). A central role for thiols in plant tolerance to abiotic stress. Int. J. Mol. Sci..

[25] Terzi R., Saruhan G.N., Güven F.G., Kadioglu A. (2018). Alpha lipoic acid treatment induces the antioxidant system and ameliorates lipid peroxidation in maize seedlings under osmotic stress. Arch. Biol. Sci..

[26] Fogacci F., Rizzo M., Krogager C., Kennedy C., Georges C.M., Knežević T., Liberopoulos E., Vallée A., Pérez-Martínez P., Wenstedt E.F. (2020). Safety evaluation of α-lipoic acid supplementation: A systematic review and meta-analysis of randomized placebo-controlled clinical studies. Antioxidants.

[27] Tibullo D., Volti G.L., Giallongo C., Grasso S., Tomassoni D., Anfuso C.D., Lupo G., Amenta F., Avola R., Bramanti V. (2017). Biochemical and clinical relevance of alpha lipoic acid: Antioxidant and anti-inflammatory activity, molecular pathways and therapeutic potential. Inflamm. Res..

[28] Packer L., Witt E.H., Tritschler H.J. (1995). Alpha-lipoic acid as a biological antioxidant. Free Radic. Biol. Med..

[29] Sezgin A., Altuntaş C., Demiralay M., Cinemre S., Terzi R. (2019). Exogenous alpha lipoic acid can stimulate photosystem II activity and the gene expressions of carbon fixation and chlorophyll metabolism enzymes in maize seedlings under drought. J. Plant Physiol..

[30] Gorcek Z., Erdal S. (2015). Lipoic acid mitigates oxidative stress and recovers metabolic distortions in salt-stressed wheat seedlings by modulating ion homeostasis, the osmo-regulator level and antioxidant system. J. Sci. Food Agric..

[31] Sgherri C., Quartacci M.F., Izzo R., Navari-Izzo F. (2002). Relation between lipoic acid and cell redox status in wheat grown in excess copper. Plant Physiol. Biochem..

[32] Álvarez C., Ángeles Bermúdez M., Romero L.C., Gotor C., García I. (2012). Cysteine homeostasis plays an essential role in plant immunity. New Phytol..

[33] Genisel M., Erdal S., Kizilkaya M. (2015). The mitigating effect of cysteine on growth inhibition in salt-stressed barley seeds is related to its own reducing capacity rather than its effects on antioxidant system. Plant Growth Regul..

[34] Rausch T., Wachter A. (2005). Sulfur metabolism: A versatile platform for launching defence operations. Trends Plant Sci..

[35] Takahashi H., Kopriva S., Giordano M., Saito K., Hell R. (2011). Sulfur assimilation in photosynthetic organisms: Molecular functions and regulations of transporters and assimilatory enzymes. Annu. Rev. Plant Biol..

[36] Terzi H., Yıldız M. (2021). Proteomic analysis reveals the role of exogenous cysteine in alleviating chromium stress in maize seedlings. Ecotoxicol. Environ. Saf..

[37] Sauter M., Moffatt B., Saechao M.C., Hell R., Wirtz M. (2013). Methionine salvage and S-adenosylmethionine: Essential links between sulfur, ethylene and polyamine biosynthesis. Biochem. J..

[38] Yang S.F., Hoffman N.E. (1984). Ethylene biosynthesis and its regulation in higher plants. Annu. Rev. Plant Physiol..

[39] Al Ubeed H., Wills R., Bowyer M., Golding J. (2019). Inhibition of postharvest senescence of green leafy vegetables by exogenous D-cysteine and L-cysteine as precursors of hydrogen sulphide. J. Hortic. Sci. Biotechnol..

[40] Allen R.G., Pereira L.S., Raes D., Smith M. (1998). Crop evapotranspiration-Guidelines for computing crop water requirements-FAO Irrigation and drainage paper 56. Fao Rome.

[41] Costache M.A., Campeanu G., Neata G. (2012). Studies concerning the extraction of chlorophyll and total carotenoids from vegetables. Rom. Biotechnol. Lett..

[42] Bates L.S., Waldren R.P., Teare I. (1973). Rapid determination of free proline for water-stress studies. Plant Soil.

[43] Chow P.S., Landhäusser S.M. (2004). A method for routine measurements of total sugar and starch content in woody plant tissues. Tree Physiol..

[44] Ünyayar S., Keleþ Y., Ünal E. (2004). Proline and ABA levels in two sunflower genotypes subjected to water stress. Bulg. J. Plant Physiol..

[45] Heath R.L., Packer L. (1968). Photoperoxidation in isolated chloroplasts: I. Kinetics and stoichiometry of fatty acid peroxidation. Arch. Biochem. Biophys..

[46] Velikova V., Yordanov I., Edreva A. (2000). Oxidative stress and some antioxidant systems in acid rain-treated bean plants: Protective role of exogenous polyamines. Plant Sci..

[47] Huang D., Ou B., Prior R.L. (2005). The chemistry behind antioxidant capacity assays. J. Agric. Food Chem..

[48] De Carvalho L.M.J., Gomes P.B., de Oliveira Godoy R.L., Pacheco S., do Monte P.H.F., de Carvalho J.L.V., Nutti M.R., Neves A.C.L., Vieira A.C.R.A., Ramos S.R.R. (2012). Total carotenoid content, α-carotene and β-carotene, of landrace pumpkins (*Cucurbita moschata* Duch): A preliminary study. Food Res. Int..

[49] Chang C.-C., Yang M.-H., Wen H.-M., Chern J.-C. (2002). Estimation of total flavonoid content in propolis by two complementary colorimetric methods. J. Food Drug Anal..

[50] Shahidi F., Naczk M., Griffiths W. (1996). Food phenolics: Sources, chemistry, effects, applications. Trends Food Sci. Technol..

[51] Association of Official Analytical Chemists (1990). Official Methods of Analysis. Association of Official Analytical Chemists. Official Method 985.33. Vitamin C, (Reduced Ascorbic Acid) in Ready-to-Feed Milk Based Infant Formula 2, 6-Dichloroindophenol Titrimetric Method.

[52] Bradford M.M. (1976). A rapid and sensitive method for the quantitation of microgram quantities of protein utilizing the principle of protein-dye binding. Anal. Biochem..

[53] Beyer F.W., Fridovich I. (1987). Assaying for superoxide dismutase activity: Some large consequences of minor changes in conditions. Anal. Biochem..

[54] Cakmak I., Strbac D., Marschner H. (1993). Activities of hydrogen peroxide-scavenging enzymes in germinating wheat seeds. J. Exp. Bot..

[55] Nakano Y., Asada K. (1981). Hydrogen peroxide is scavenged by ascorbate-specific peroxidase in spinach chloroplasts. Plant Cell Physiol..

[56] Dias M.A., Costa M.M. (1983). Effect of low salt concentrations on nitrate reductase and peroxidase of sugar beet leaves. J. Exp. Bot..

[57] American Association of Cereal Chemists (2012). International Methods Approved of the American Association of Cereal Chemists.

[58] Association of Official Analytical Chemists (2012). Official Methods of the Association of Official Analytical Chemists.

[59] Snedecor G.W., Cochran W.G. (1967). Statistical Methods.

[60] Rousseeuw P.J. (1987). Silhouettes: A graphical aid to the interpretation and validation of cluster analysis. J. Comput. Appl. Math..

[61] Wickelmaier F. (2003). An Introduction to MDS.

[62] Mihailović N., Lazarević M., Dželetović Z., Vučković M., Đurđević M. (1997). Chlorophyllase activity in wheat, *Triticum aestivum* L. leaves during drought and its dependence on the nitrogen ion form applied. Plant Sci..

[63] Hassan N., Ebeed H., Aljaarany A. (2020). Exogenous application of spermine and putrescine mitigate adversities of drought stress in wheat by protecting membranes and chloroplast ultra-structure. Physiol. Mol. Biol. Plants.

[64] Ibrahim M.F.M., Ibrahim H.A., Abd El-Gawad H.G. (2021). Folic acid as a protective agent in snap bean plants under water deficit conditions. J. Hortic. Sci. Biotechnol..

[65] Xu H., Vavilin D., Vermaas W. (2001). Chlorophyll b can serve as the major pigment in functional photosystem II complexes of cyanobacteria. Proc. Natl. Acad. Sci. USA.

[66] Hoober J.K. (2016). The Role of Chlorophyll b in Photosynthesis: Hypothesis. Photosynthesis.

[67] Perveen S., Iqbal M., Saeed M., Iqbal N., Zafar S., Mumtaz T. (2019). Cysteine-induced alterations in physicochemical parameters of oat (*Avena sativa* L. var. Scott and F-411) under drought stress. Biol. Futur..

[68] Sadak M.S., Abd El-Hameid A.R., Zaki F.S., Dawood M.G., El-Awadi M.E. (2020). Physiological and biochemical responses of soybean (*Glycine max* L.) to cysteine application under sea salt stress. Bull. Natl. Res. Cent..

[69] Ibrahim M. (2014). Induced drought resistance in common bean (*Phaseolus vulgaris* L.) by exogenous application with active yeast suspension. Middle East J. Appl. Sci..

[70] Abd El-Gawad H.G., Mukherjee S., Farag R., Abd Elbar O.H., Hikal M.S., Abou El-Yazied A., Abd Elhady S.A., Helal N.A., Elkelish A., El Nahhas N. (2020). Exogenous γ-aminobutyric acid (GABA)-induced signaling events and field performance associated with mitigation of drought stress in *Phaseolus vulgaris* L.. Plant Signal. Behav..

[71] Abd Elhady S.A., El-Gawad H.G.A., Ibrahim M.F., Mukherjee S., Elkelish A., Azab E., Gobouri A.A., Farag R., Ibrahim H.A., El-Azm N.A. (2021). Hydrogen peroxide supplementation in irrigation water alleviates drought stress and boosts growth and productivity of potato plants. Sustainability.

[72] Hasan M.M., Rahman M.A., Skalicky M., Alabdallah N.M., Waseem M., Jahan M.S., Ahammed G.J., El-Mogy M.M., El-Yazied A.A., Ibrahim M.F.M. (2021). Ozone Induced Stomatal Regulations, MAPK and Phytohormone Signaling in Plants. Int. J. Mol. Sci..

[73] Turk H., Erdal S., Karayel U., Dumlupinar R. (2018). Attenuation of lead toxicity by promotion of tolerance mechanism in wheat roots by lipoic acid. Cereal Res. Commun..

[74] Parry A.D., Horgan R. (1991). Carotenoids and abscisic acid (ABA) biosynthesis in higher plants. Physiol. Plant..

[75] Rolczynski B.S., Navotnaya P., Sussman H.R., Engel G.S. (2016). Cysteine-mediated mechanism disrupts energy transfer to prevent photooxidation. Proc. Natl. Acad. Sci. USA.

[76] Akula R., Ravishankar G.A. (2011). Influence of abiotic stress signals on secondary metabolites in plants. Plant Signal. Behav..

[77] Nakabayashi R., Yonekura-Sakakibara K., Urano K., Suzuki M., Yamada Y., Nishizawa T., Matsuda F., Kojima M., Sakakibara H., Shinozaki K. (2014). Enhancement of oxidative and drought tolerance in Arabidopsis by overaccumulation of antioxidant flavonoids. Plant J..

[78] Weidner S., Karolak M., Karamac M., Kosinska A., Amarowicz R. (2009). Phenolic compounds and properties of antioxidants in grapevine roots [*Vitis vinifera* L.] under drought stress followed by recovery. Acta Soc. Bot. Pol..

[79] Falcone Ferreyra M.L., Rius S., Casati P. (2012). Flavonoids: Biosynthesis, biological functions, and biotechnological applications. Front. Plant Sci..

[80] Baier M., Dietz K.-J. (1999). Protective function of chloroplast 2-cysteine peroxiredoxin in photosynthesis. Evidence from transgenic Arabidopsis. Plant Physiol..

[81] Abuelsoud W., Hirschmann F., Papenbrock J. (2016). Sulfur metabolism and drought stress tolerance in plants. Drought Stress Tolerance in Plants.

[82] Alnusairi G.S., Mazrou Y.S., Qari S.H., Elkelish A.A., Soliman M.H., Eweis M., Abdelaal K., El-Samad G.A., Ibrahim M.F.M., ElNahhas N. (2021). Exogenous Nitric Oxide Reinforces Photosynthetic Efficiency, Osmolyte, Mineral Uptake, Antioxidant, Expression of Stress-Responsive Genes and Ameliorates the Effects of Salinity Stress in Wheat. Plants.

[83] Nahhas N.E., Abdelaal K.A., AlKahtani M.D., al Husnain L., AlGwaiz H.I., Hafez Y.M., Attia K.A., El-Esawi M.A., Ibrahim M.F., Elkelish A. (2021). Biochar and jasmonic acid application attenuate antioxidative systems and improves growth, physiology, nutrient uptake and productivity of faba bean (*Vicia faba* L.) irrigated with saline water. Plant Physiol. Biochem..

[84] Gupta N., Gupta S., Kumar A. (2001). Effect of water stress on physiological attributes and their relationship with growth and yield of wheat cultivars at different stages. J. Agron. Crop Sci..

[85] Zhao W., Liu L., Shen Q., Yang J., Han X., Tian F., Wu J. (2020). Effects of water stress on photosynthesis, yield, and water use efficiency in winter wheat. Water.

[86] Li G., Gao H., Zhao B., Dong S., Zhang J., Yang J., Wang J., Liu P. (2009). Effects of drought stress on activity of photosystems in leaves of maize at grain filling stage. Acta Agron. Sin..

[87] Barutçular C., Dizlek H., El-Sabagh A., Sahin T., EL-Sabagh M., Islam M.S. (2016). Nutritional quality of maize in response to drought stress during grain-filling stages in Mediterranean climate condition. J. Exp. Biol. Agric. Sci..

[88] Hammad S.A., Ali O.A. (2014). Physiological and biochemical studies on drought tolerance of wheat plants by application of amino acids and yeast extract. Ann. Agric. Sci..

[89] Xiao R., Wang X., Jiang L., Tang H. (2018). Research and Application of Lipoic Acid in Plants. IOP Conf. Ser. Earth Environ. Sci..

[90] Guan X., Okazaki Y., Zhang R., Saito K., Nikolau B.J. (2020). Dual-localized enzymatic components constitute the fatty acid synthase systems in mitochondria and plastids. Plant Physiol..

[91] Mukwevho E., Ferreira Z., Ayeleso A. (2014). Potential role of sulfur-containing antioxidant systems in highly oxidative environments. Molecules.

[92] Hasanuzzaman M., Bhuyan M., Mahmud J., Nahar K., Mohsin S., Parvin K., Fujita M. (2018). Interaction of sulfur with phytohormones and signaling molecules in conferring abiotic stress tolerance to plants. Plant Signal. Behav..

[93] Alzahrani O., Abouseadaa H., Abdelmoneim T.K., Alshehri M.A., El-beltagi H.S., El-Mogy M.M., Atia M.A.M. (2021). Agronomical, physiological and molecular evaluation reveals superior salt-tolerance in bread wheat through salt-induced priming approach. Not. Bot. Horti Agrobot. Cluj Napoca.

[94] Hruskova M., Smejda P. (2003). Wheat flour dough alveograph characteristics predicted by NIR Systems 6500. Czech J. Food Sci..

