# Supplementary material for: Roles of Exogenous α-Lipoic Acid and Cysteine in Mitigation of Drought Stress and Restoration of Grain Quality in Wheat
Source: Plants (Basel). 2021 Oct 28;10(11):2318. doi: 10.3390/plants10112318 (PMC8619972; doi:10.3390/plants10112318)
Supplement: Supplementary file 1 [file plants-10-02318-s001.zip › plants-1397016-supplementary.pdf]

Table S1: Germination assay of Wheat seed after soaking with different concentration of α-lipoic acid

ALA	Time 2 h	4h	8 h
**Fresh weight (mg. seedling $^{-1}$)**			
**Control**	52.08 ± 0.28 de	54.48 ± 0.71 cd	54.23 ±0.66 cd
**1 µM**	54.51 ± 0.40 cd	55.02 ± 0.95 cd	47.93 ± 0.08 f
**5 µM**	55.65 ± 1.48 cd	56.54 ± 0.42 bc	48.04 ± 0.09 f
**10 µM**	54.77 ± 0.69 cd	59.86 ± 0.79 b	46.13 ± 0.56 fgh
**20 µM**	55.87 ± 1.39 cd	66.49 ± 1.19 a	42.41 ± 1.29 hi
**50 µM**	49.80 ± 1.03 ef	46.59 ± 1.74 fg	37.30 ± 0.64 j
**100 µM**	43.89 ± 0.84 ghi	41.31 ± 0.48 i	34.31 ± 1.60 j
**Antioxidant capacity by DPPH assay (%)**			
**Control**	66.78 ± 0.71 fg	73.47 ± 0.79 d	67.97 ± 0.11 ef
**1 µM**	68.51 ± 0.36 ef	74.53 ± 2.03 cd	62.26 ± 0.67 hi
**5 µM**	67.93 ± 0.47 ef	77.38 ± 0.80 bc	58.62 ± 0.52 ijk
**10 µM**	68.14 ± 0.82 ef	79.50 ± 0.87 b	56.37 ± 1.08 jk
**20 µM**	71.62 ± 1.61 de	86.99 ± 1.44 a	55.93 ± 0.90 jk
**50 µM**	63.64 ± 0.76 gh	58.80 ± 0.92 ij	45.80 ± 1.32 m
**100 µM**	54.68 ± 1.13 kl	51.05 ± 0.34 l	40.88 ± 0.62 n
